# Comparative genome analyses of clinical and non-clinical *Clostridioides difficile* strains

**DOI:** 10.3389/fmicb.2024.1404491

**Published:** 2024-06-27

**Authors:** Miriam A. Schüler, Thomas Riedel, Jörg Overmann, Rolf Daniel, Anja Poehlein

**Affiliations:** ^1^Genomic and Applied Microbiology and Göttingen Genomics Laboratory, Institute of Microbiology and Genetics, Georg-August-University, Göttingen, Germany; ^2^Leibniz Institute DSMZ-German Collection of Microorganisms and Cell Cultures, Braunschweig, Germany; ^3^German Center for Infection Research (DZIF), Partner Site Braunschweig-Hannover, Braunschweig, Germany; ^4^Institute of Microbiology, Technische Universität Braunschweig, Braunschweig, Germany

**Keywords:** *Clostridioide difficile*, virulence, mobile genetic element, clinical, non-clinical, genome comparison

## Abstract

The pathogenic bacterium *Clostridioides difficile* is a worldwide health burden with increasing morbidity, mortality and antibiotic resistances. Therefore, extensive research efforts are made to unravel its virulence and dissemination. One crucial aspect for *C. difficile* is its mobilome, which for instance allows the spread of antibiotic resistance genes (ARG) or influence strain virulence. As a nosocomial pathogen, the majority of strains analyzed originated from clinical environments and infected individuals. Nevertheless, *C. difficile* can also be present in human intestines without disease development or occur in diverse environmental habitats such as puddle water and soil, from which several strains could already be isolated. We therefore performed comprehensive genome comparisons of closely related clinical and non-clinical strains to identify the effects of the clinical background. Analyses included the prediction of virulence factors, ARGs, mobile genetic elements (MGEs), and detailed examinations of the pan genome. Clinical-related trends were thereby observed. While no significant differences were identified in fundamental *C. difficile* virulence factors, the clinical strains carried more ARGs and MGEs, and possessed a larger accessory genome. Detailed inspection of accessory genes revealed higher abundance of genes with unknown function, transcription-associated, or recombination-related activity. Accessory genes of these functions were already highlighted in other studies in association with higher strain virulence. This specific trend might allow the strains to react more efficiently on changing environmental conditions in the human host such as emerging stress factors, and potentially increase strain survival, colonization, and strain virulence. These findings indicated an adaptation of the strains to the clinical environment. Further, implementation of the analysis results in pairwise genome comparisons revealed that the majority of these accessory genes were encoded on predicted MGEs, shedding further light on the mobile genome of *C. difficile*. We therefore encourage the inclusion of non-clinical strains in comparative analyses.

## 1 Introduction

The bacterium *Clostridioides difficile* is a globally widespread pathogen that constitutes a major cause of nosocomial and antibiotic-associated infections, with disease severity ranging from mild diarrhea to pseudomembranous colitis, eventually leading to death (Balsells et al., 2019). A *C. difficile* infection is mainly elicited after antibiotic treatment and increasing antibiotic resistances in this species impede successful treatment of an infection (Spigaglia, 2016). *C. difficile* is extensively studied, especially in the context of increasing multi-drug resistances, but also concerning its virulence heterogeneity. *C. difficile* strains can extremely vary in the induced symptoms, and even non-toxigenic strains without disease-causing toxins exist (Czepiel et al., 2019). Research on *C. difficile* virulence already pointed towards the importance of mobile genetic elements (MGE). About 11% of a *C. difficile* genome is composed of MGEs, including plasmids, bacteriophages, IS elements, and conjugative and mobilizable transposons (Sebaihia et al., 2006; Mullany et al., 2015). Plasmids can contribute to virulence by carrying toxin genes or promoting antibiotic resistances (Smits et al., 2022), and also bacteriophages can influence *C. difficile* virulence (Govind et al., 2009; Sekulovic et al., 2011; Goh et al., 2013; Riedel et al., 2017b; Mehner-Breitfeld et al., 2018). MGEs are especially crucial for horizontal gene transfer that allows fast adaptation to environmental conditions, e.g., spreading genes conferring antibiotic resistances between different strains or even species (de la Cruz and Davies, 2000). In addition, the pathogenicity locus of *C. difficile*, which encodes the *C. difficile*-typical toxin genes, exhibits a mobile character and can transfer to a previously non-toxigenic strain (Brouwer et al., 2013). Although the toxin genes represent the major virulence factors of *C. difficile*, their contribution to overall virulence is still under debate, and other aspects such as tolerance to secondary bile acids or specific accessory genes rather correlated with disease severity (Lewis et al., 2017).

As a prominent pathogen with increasing morbidity and mortality, most of the analyzed *C. difficile* strains originate from clinical specimen of infected individuals. However, *C. difficile* was also found in asymptomatic, healthy individuals, and is also a natural inhabitant of various animal species and environmental reservoirs (Ozaki et al., 2004; Janezic et al., 2016; Weese, 2020). Although several *C. difficile* strains were isolated from diverse environmental sources in recent years, genome-based comparisons always comprised strains associated with infection (“clinical”) or only worked on draft genomes and also rather focused on epidemiological studies (Knight et al., 2017; Xu et al., 2021; Zhou et al., 2021; Dong et al., 2023). Zhou et al. (2021) compared strains of environmental or clinical origin and could not observe a connection to the isolation source. However, their analyses did not comprise pan-genomic studies but focused on virulence factors and antibiotic resistance genes (ARG). Comprehensive pan-genomic analyses specifically comparing clinical and non-clinical strains have not been conducted, yet.

In this study, we performed genomic analyses between high quality genomes of *C. difficile* strains originating from environmental samples (non-clinical background) and from infected humans (clinical reference strains). Analyses were compared between clinical and non-clinical strain corresponding in their sequence type. We focused on MGEs and potentially linked genes encoding antibiotic resistances or virulence factors, and conducted a pan genome analysis. All these analyses were put into genomic context through direct genome comparisons of the corresponding clinical and non-clinical strains. We detected genomic differences that were linked to clinical background and might reflect increased physiological adaptation ability.

## 2 Methods

### 2.1 Strain isolation and cultivation

Non-clinical *C. difficile* strains were isolated from horse feces, biogas fermenter sludge and mud. The environmental samples were collected between November 2019 and July 2020 with sterile canonical falcon tubes and were stored at 4°C upon arrival in the laboratory. We opted for antibiotic-free isolation to overcome a potential isolation bias (Schüler et al., 2023). Different antibiotic-free and antibiotic-based isolation approaches were therefore employed. As a result, strains J2_1 and TS3_3 were isolated without antibiotics, whereas MA_1 and B1_2 originated from isolation approaches with moxalactam norfloxacin (CDMN, Oxoid Deutschland GmbH, Wesel, Germany). Details of isolation protocols are described in Supplementary Presentation 1. In general, environmental samples were dissolved in anoxic PBS (pH 7.4) [Phosphate-buffered saline (PBS), 2006] and pasteurized before inoculating the enrichment media. Grown enrichment cultures were plated on solid media with 1.5% agar and colonies examined for identity via 16S rRNA gene Sanger sequencing using colony PCR with Phusion High-Fidelity polymerase (Thermo Fisher Scientific, Waltham, MA, USA) and primers 08f (5′-AGAGTTTGATCCTGGC-3′) and 1504r (5′-TACCTTGTTACGACTT-3′), following the recommendations of the manufacturer. PCR products were purified with the QIAquick PCR Purification kit (Qiagen, Hilden, Germany) as recommended by the manufacturer and subjected to Sanger sequencing by Microsynth Seqlab GmbH (Göttingen, Germany).

Clinical reference strains of sequence types (ST)/ribotypes (RT) corresponding to the four non-clinical strains were kindly provided by the Institute of Medical Microbiology, Göttingen, Germany. Strains DSM 28196, DSM 29747, SC083-01-01, and SC084-01-01 had been isolated from infected humans as described in Riedel et al. (2017a).

Isolates were routinely cultivated at 37°C under anoxic conditions in supplemented Brain Heart Infusion Broth (BHIS; supplemented with 0.5% yeast extract, 0.05% L-cysteine, 0.0001% Na-resazurin, purged with nitrogen).

### 2.2 DNA extraction

Genomic DNA was extracted from overnight cultures using the MasterPure Gram Positive DNA Purification kit as recommended by the manufacturer (Epicentre, Madison, WI, United States). DNA quality was assessed on a NanoDrop ND-1000 (Peqlab Biotechnologie GmbH, Erlangen, Germany), and DNA concentration was measured using the Qubit 3.0 Fluorometer (Thermo Fisher Scientific) with the BR dsDNA assay kit.

### 2.3 Ribotyping of *Clostridioides difficile* isolates

Isolated strains were phylogenetically examined via ribotyping based on Bidet et al. (1999). Amplification of the 16S-23S rRNA intergenic spacer region was conducted with the Dreamtaq polymerase (Thermo Fisher Scientific) using reagents as recommended by the manufacturer with 0.2 mM of each primer and 50 ng template DNA per 50 μL PCR reaction. PCR cycling comprised initial denaturation at 95°C for 3 min, followed by 30 cycles of 95°C for 1 min, 56°C for 30 s, and 72°C for 1 min. Final elongation was performed at 72°C for 5 min. PCR products were separated on a 2% agarose gel ran at 5 V/cm with subsequent staining using ethidium bromide and visualization with the AlphaImager HP (Alpha Innotech Corp., San Leandro, USA) and AlphaView Software (v3.5.0). For RT assignment, observed band patterns were compared to already known RTs.

### 2.4 Genome sequencing, assembly, and annotation

For whole-genome sequencing of the non-clinical isolates, genomic DNA was subjected to short-read and long-read sequencing using Illumina and Oxford Nanopore technology, respectively. Illumina sequencing libraries were prepared with the Nextera XT DNA sample preparation kit and sequenced using a MiSeq instrument and reagent kit v3 (2 × 300 bp, 600 cycles) as recommended by the manufacturer (Illumina, San Diego, CA, USA). For Nanopore sequencing, genomic DNA without specific size selection was processed using the ligation sequencing kit 1D (SQK-LSK109) and the native barcode expansion kit (EXP-NBD104) according to the manufacturer’s specifications (Oxford Nanopore Technologies, Oxford, United Kingdom). Nanopore sequencing was performed with the MinION system using a SpotON flow cell Mk I (R9.4.0) for 72 h. All following software was used with default settings unless otherwise stated. The MinKNOW software (v19.12.5) with implemented Guppy (v3.2.10) was used in fast mode for demultiplexing and base calling. Nanopore reads were first trimmed using Porechop (v0.2.4)^1^ and filtered with Filtlong (v0.2.1),^2^ following assembly with Flye (v2.9.2) (Kolmogorov et al., 2019). Illumina reads were processed with fastp (v0.23.3) (Chen, 2023) and trimmed using Trimmomatic (v0.39) (Bolger et al., 2014). The long-read assembly was polished with the processed short reads using softwares BWA (v0.7.17, r1188) (Li and Durbin, 2010) and Polypolish (v0.5.0) (Wick and Holt, 2022). Circularization of the assemblies were verified with Bandage v0.8.1 (Wick et al., 2015) and assemblies rotated with Circlator (v1.5.5) (Hunt et al., 2015) to begin with the gene *dnaA*. The assembled genome sequences were annotated with Prokka (v1.14.5) (Seemann, 2014). Selenoproteins were curated manually.

Genome sequencing and assembly of the clinical reference strains was done by Leibniz Institute DSMZ-German Collection of Microorganisms and Cell Cultures, Braunschweig, Germany. High molecular weight DNA was prepared using the Qiagen Genomic Tip/100 G kit (Qiagen, Hilden, Germany). SMRTbell template libraries were prepared according to the instructions from Pacific Biosciences, Menlo Park, CA, United States, following the Procedure & Checklist - 20 kb Template Preparation Using BluePippin Size-Selection System. Briefly, for preparation of 15 kb libraries 5 μg genomic DNA were end-repaired and ligated overnight to hairpin adapters applying components from the DNA/Polymerase Binding Kit P6 from Pacific Biosciences, Menlo Park, CA, United States. Reactions were carried out according to the manufacturer’s instructions. BluePippin Size-Selection was performed according to the manufacturer’s instructions (Sage Science, Beverly, MA, United States). Conditions for annealing of sequencing primers and binding of polymerase to purified SMRTbell template were assessed with the Calculator in RS Remote, PacificBiosciences, Menlo Park, CA, United States. SMRT sequencing was carried out on the PacBio RSII (PacificBiosciences, Menlo Park, CA, United States) taking 240-min movies. Long read genome assembly was performed with the “RS_HGAP_Assembly.3 “protocol included in SMRTPortal (v2.3.0) using default parameters. Chromosomal contigs and plasmids were circularized, particularly artificial redundancies at the ends of the contigs were removed and adjusted to *dnaA*. Identification of redundancies and the replication genes has been done based on BLAST, circularization and rotation to the replication genes has been performed by genomecirculator.jar tool.^3^ Error-correction was performed by a mapping of Illumina short reads onto finished genome using BWA (v0.6.2) in paired-end (sampe) mode using default settings (Li and Durbin, 2010) with subsequent variant and consensus calling using VarScan (v2.3.6) (Koboldt et al., 2012).

### 2.5 Genomic analyses

In general, plots were created with RStudio (v2022.06.0) (RStudio Team, 2020) using the package ggplot2 (v3.4.2) (Wickham, 2016), and final modifications were done with Inkscape (v0.48).^4^

MLST assignment of the non-clinical strains was done using PubMLST (Jolley et al., 2018). Genome qualities were assessed with CheckM2 (v1.0.2) (Chklovski et al., 2023) before performing genome analyses.

The program antiSMASH (v7.0.0) (Blin et al., 2023) was used for predicting secondary metabolite biosynthetic gene clusters. Putative ARGs were identified with RGI (v6.0.2), CARD (v3.2.7) (Alcock et al., 2023), and AMRFinderPlus (v3.11.14) (Feldgarden et al., 2021) employing the NCBI Bacterial Antimicrobial Resistance Reference Gene Database (v2023-07-13.2).

Screening for virulence factors was performed by BLAST+ blastp analysis (v2.12.0) (Camacho et al., 2009) (options: -num_alignment 1; −outfmt “6 delim=, qaccver saccver pident length evalue qcovs qcovhsp bitscore”) using the *C. difficile-*associated protein sequences present in the full dataset (retrieved on 14.07.2023) from the virulence factor database (VFDB (Liu et al., 2022)) as query against the whole-genome protein sequences of the analyzed strains. The *spo0A* sporulation gene from *C. difficile* strain 630 was additionally included in the analysis (CP010905.2, CDIF630_01363). Protein sequences of each virulence factor between corresponding genomes were additionally compared with blastp (Camacho et al., 2009) (options like above) to check for sequence deviations.

Presence/absence of ARGs and protein sequence query coverage /percentage identity of virulence factors was visualized as heatmaps.

After initial assessment of toxin gene presence with the aforementioned VFDB analysis, the corresponding toxin-operons and adjacent genes were inspected for nucleotide sequence similarity and genomic location by sequence alignment with clinker (v1.32) (Gilchrist and Chooi, 2021), including reference sequences from *C. difficile* strain 630 (CP010905.2, CDIF630_00771–00782) and R20291 (CP029423.1, CDIF27147_02765–02770), respectively.

#### 2.5.1 Analysis of MGEs

Genomes were analyzed with PlasmidFinder (v2.1) (Carattoli et al., 2014) for plasmid family identification. Insertion sequences (IS) were identified with ISEScan (v1.7.2.3) (Xie and Tang, 2017). Genomic islands (GI) were predicted with various tools, including PHASTEST in deep mode (on 25.6.23) (Wishart et al., 2023) for prophage prediction, IslandViewer 4 (accessed on 4.7.23) (Bertelli et al., 2017) and ICEscreen (v1.2.0) (Lao et al., 2022). The numbers of identified MGEs and their types were visualized as heatmaps.

#### 2.5.2 Pan genome analysis

The core/pan genome including all genomes was calculated with Roary (v3.13.0) (Page et al., 2015), and a Venn diagram visualizing the results was created with Inkscape (v0.48)^4^ Venn diagrams showing the shared and unique genes for each pair of non-clinical and corresponding clinical strain as estimated by Roary were visualized with ggplot2. The unique genes were assigned to functional clusters of orthologous groups of proteins (COG) with eggNOG-mapper (v2.1.9) (Cantalapiedra et al., 2021). Relative abundance of unique genes of a specified COG was determined for each genome relative to its total number of coding sequences (CDS) and visualized as bar charts. Further, the differences between clinical and non-clinical strain of these relative COG-gene abundances were calculated by subtracting the relative values of the non-clinical from the clinical strain. These difference values were also plotted.

#### 2.5.3 Pairwise genome alignment and comparison

For direct genome comparison, genomes of non-clinical and corresponding clinical reference strains were first aligned with Mauve (v20150226) (Darling et al., 2004) and inspected for significant sequence deviations detected as alignment gaps over multiple CDSs. These CDSs were inspected for their predicted function and compared to the previous pan genome analysis. Further, Proksee (specifically: CGView Builder v1.1.2 + Features v1.0.0) (Grant et al., 2023) was used for visualization of each genome complemented with its previously predicted ARGs, MGEs and unique genes. Additionally, pairwise genome alignments of clinical and non-clinical strains were performed with MUMmer (v3.23) (Kurtz et al., 2004) (options: -maxmatch; −l 100; −b), with each genome used as query. The resulting alignment positions at the reference sequence were also implemented in the genome visualization. Proksee depictions of corresponding genomes were combined and modified using Inkscape (v0.48) for direct genome comparison.

## 3 Results and discussion

### 3.1 General genome characteristics

The four non-clinical *C. difficile* strains belong to ST1/RT027 (*C. difficile* TS3_3), ST3/RT001/072 (*C. difficile* B1_2), ST8/RT002 (*C. difficile* J2_1), and ST11/RT078 (*C. difficile* MA_1). These STs/RTs are known for their high clinical relevance and/or prevalence and prominent representatives of the phylogenetic clades (Table 1) (Walker et al., 2013; Knight et al., 2021). Strains ST1/RT027 and ST11/RT078 are further prominent representatives of *C. difficile* strains that carry genes for the binary toxins additionally to the *C. difficile*-typical toxins (Martínez-Meléndez et al., 2022). Clinical strains DSM 28196 (ST1/RT027), SC084-01-01 (ST3/RT001/072), SC083-01-01 (ST8/RT002), and DSM 29747 (ST11/RT078) corresponding in ST/RT to the non-clinical strains were used in genome-based investigations covering analyses of MGEs as well as core and accessory genes. Throughout this work, the mentioning of corresponding strains refers to clinical and non-clinical strains of the same ST.

**Table 1:** General genomic features of the analyzed strains.

Non-clinical *C. difficile* strains				
Strain name	TS3_3	B1_2	J2_1	MA_1
ST / clade	1/2	3/1	8/1	11/5
Designation	ST1-env	ST3-env	ST8-env	ST11-env
RT	027	001/072	002	078
GenBank accession	CP134872	CP132141 CP132142 CP132143	CP134690 CP134691	CP132139 CP132140
Chromosome size (bp)	4,116,134	4,194,230	4,081,925	3,970,170
ECEs size (bp)	-	42,358 / 7,624	46,261	33,670
No. of CDS	3,630	3,763	3,652	3,545
No. of curated selenoproteins	4	4	4	4
No. of rRNA	35	35	35	35
No. of tRNA	90	90	90	90
Clinical *C. difficile* strains				
Strain name	DSM 28196	SC084-01-01^a^	SC083-01-01^a^	DSM 29747
ST / clade	1 / 2	3 / 1	8 / 1	11 / 5
Designation	ST1-med	ST3-med	ST8-med	ST11-med
RT	027	001/072	002	078
GenBank accession	CP012320	CP132146 CP132147 CP132148	CP132144 CP132145	CP019864
Chromosome size (bp)	4,205,365	4,184,644	4,122,919	4,071,596
ECEs size (bp)	-	47,363 / 130,799	45,313	-
No. of CDS	3,707	3,950	3,704	3,556
No. of curated selenoproteins	4	4	4	4
No. of rRNA	35	34	35	35
No. of tRNA	91	92	90	90

All eight genomes were initially evaluated for quality. The CheckM2 analysis thoroughly verified uniform genome completeness (99.86–99.99%) and purity (0.1–0.78% contamination).

General genomic features of the analyzed strains are listed in Table 1. In the following, the analyzed strains/genomes will be designated with their ST and clinical background (clinical = med, non-clinical = env) instead of their actual strain name (see Table 1). Most of the genomes comprised extrachromosomal elements (ECE). The ST3 genomes exhibited even two co-occurring ECEs. However, ECE carriage was not necessarily linked to the ST. Additionally, ECE size varied between the genomes of corresponding clinical and non-clinical strains, indicating their divergence. The clinical strains exhibited larger total genome size (including ECEs), and correspondingly more CDSs than non-clinical strains. No differences between clinical and non-clinical strains were recorded with respect to the number of rRNA and tRNA genes.

The screening for putative gene clusters encoding biosynthesis of secondary metabolites did not show differences between corresponding strains. All strains possessed regions predicted to encode cyclic-lactone-autoinducer, non-ribosomal peptide synthetase, or ranthipeptide. Since the capacity for secondary metabolite production did not differ between the strains, they were not considered in further analyses.

### 3.2 *In silico* examination of virulence factors for genomic assessment of virulence potential

**Figure 1:**
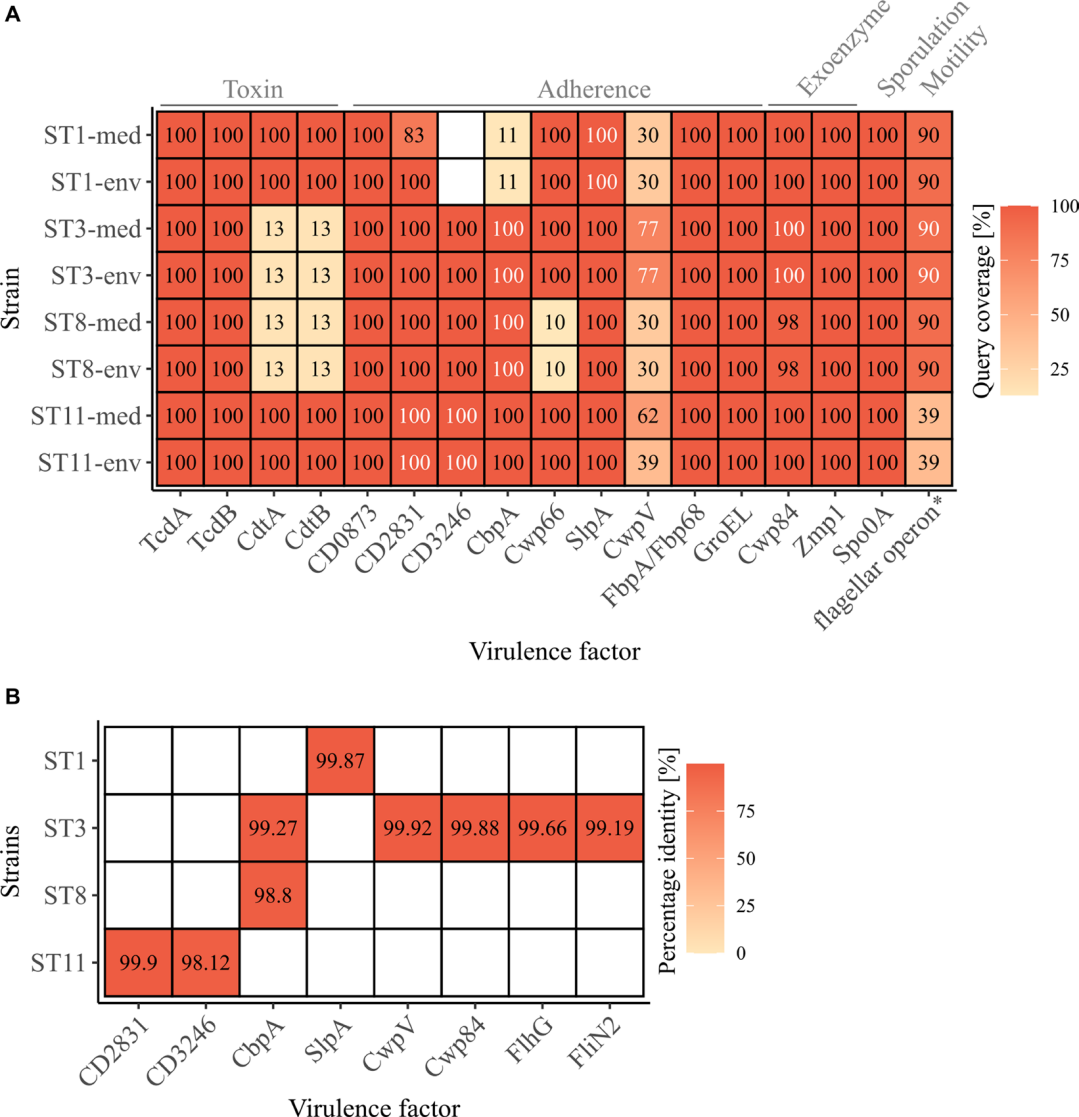
*C. difficile*-associated virulence factors in the analyzed strains. **(A)** Presence of the examined virulence factors is indicated as the protein sequence query coverage to the reference VFDB dataset, by color and stated coverage value. White coverage values highlight deviating sequences in proteins of the same query coverage between clinical and non-clinical strain. Virulence factors are labelled with their names as obtained from the VFDB dataset, and their related functions are stated on top. *flagellar operon comprising 41 CDSs obtained from the VFDB dataset, and its query coverage calculated as the relative number of present CDSs of the total 41. **(B)** Sequence similarity as percentage identity between clinical and non-clinical strains of the proteins highlighted in **(A)** as white.

**Figure 2:**
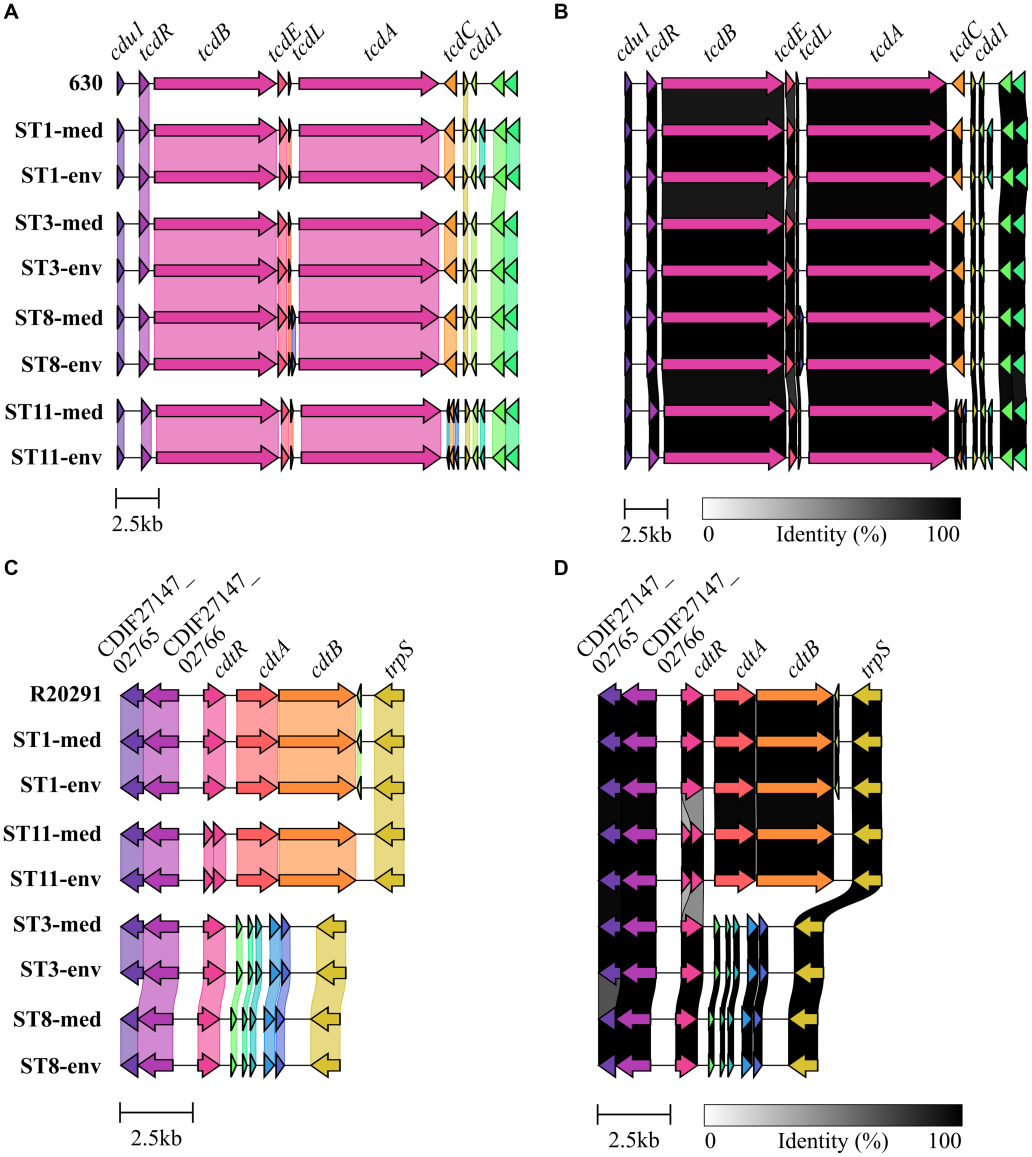
Gene cluster comparisons of toxin loci. Genes within and next to the toxin-encoding loci were compared on nucleotide sequence level between all analyzed strains for **(A,B)** the PaLoc with 630 as reference, and **(C,D)** the CdtLoc with R20291 as reference sequence. **(A,C)** depict 100% sequence identity, while **(B,D)** represent identities above 80%.

Genomic examinations of the strains for the presence of fundamental virulence factors of *C. difficile* (listed in VFDB (Liu et al., 2022)) with BLAST+ blastp (Camacho et al., 2009) were performed to assess the virulence potential of the corresponding strains. Thereby, protein sequences of the virulence factors were compared to the reference sequence to define the query coverage (Figure 1A), and were further analyzed for sequence deviations between corresponding clinical and non-clinical strains (Figures 1A,B). Most of the characterized *C. difficile* virulence factors were present in the genomes of all strains. The main virulence factors in *C. difficile* pathogenicity, the disease-causing toxin genes *tcdA* and *tcdB* encoded by the pathogenicity locus (PaLoc), were identified in all strains (Figure 1A). Closer investigations of the PaLoc in all genomes including the regulatory genes *tcdC* and *tcdR*, along with *tcdE* (Mani and Dupuy, 2001; Matamouros et al., 2007; Govind and Dupuy, 2012) confirmed its consistent genomic location between the same genes (*cdu1 and cdd1*) like in the reference genome of *C. difficile* strain 630 (Monot et al., 2015) (Figures 2A,B). The recently described gene *tcdL* was also identified next to *tcdE* in all strains (Mehner-Breitfeld et al., 2018). DNA alignment of the PaLoc-operons demonstrated that genes were 100% identical between corresponding clinical and non-clinical strains and even between strains of different STs (Figure 2A), and also intergenic sequences of the PaLoc were verified to be identical among corresponding strains via BLAST analysis. All genes except *tcdE* shared at least 80% nucleotide sequence similarity to all other aligned genomes (Figure 2B). In addition to the PaLoc, another toxin-harboring locus (CdtLoc) is known in certain *C. difficile* strains of clades 2 (e.g., ST1/RT027), 3 and 5 (e.g., ST11/RT078) (Martínez-Meléndez et al., 2022), which harbors the binary toxin CDT encoded by the genes *cdtA* and *cdtB* (Gerding et al., 2014). The entire CDT genes were identified in both clinical and non-clinical strains of ST1 and ST11, while only 13% of the gene sequences were present in the other strains (Figure 1A). The CdtLoc was also found at a consistent genomic location between the same genes (CDIF27147_02765 and *trpS*) in accordance with the reference genome of *C. difficile* strain R20291 (Wang et al., 2022) (Figures 2C,D). Moreover, the regulatory gene *cdtR* was observed in all genomes. The *cdtR* gene sequences were identical in most of the genomes (Figure 2C). Strains without genes *cdtA* and *cdtB* harbored the same five small CDS instead (Figures 2C,D). In summary, toxin gene analyses confirmed uniform presence and location of toxin genes among corresponding clinical and non-clinical strains as well as identical toxin gene sequences. Sequence variants of toxin genes or the corresponding regulatory genes were demonstrated to influence strain virulence (Lanis et al., 2013; Dong et al., 2023). Based on our sequence comparisons, the corresponding clinical and non-clinical strains exhibit the same genomic virulence potential.

In addition to the toxin genes, other proteins are relevant for *C. difficile* pathogenicity, such as genes involved in cell adherence that are crucial for biofilm formation, which in turn affects resistance to harmful substances like antibiotics (Dapa and Unnikrishnan, 2013). Further important virulence factors are exoenzymes, sporulation and motility. All these virulence factors together determine the colonization efficiency (Awad et al., 2014). The majority of these virulence factors among the *C. difficile-*specific proteins were identified in the analyzed strains with 100% query coverage to the VFDB reference sequences, and only few proteins were only partially present (CbpA in ST1 strains, CwpV in all strains, Cwp66 in ST8 strains, flagellar operon in ST11 strains) or missing (CD3246 in ST1 strains) (Figure 1A). Thereby, the query coverage of the virulence factors with regard to the VFDB references was largely identical between the corresponding strains except for ST1-CD2831 and ST11-CwpV. In some cases, the protein sequences deviated between the corresponding strains by only 0.10 to 1.88% (Figures 1A,B), which often involved only one amino acid. The bigger sequence deviations were found in protein CbpA of ST3 and ST8 strains at different regions and in CD3246 of ST11 strains and consisted of a missing stretch of several amino acids (9–11 amino acids). These protein differences were detected in strains of both clinical/environmental background with regard to the VFDB reference. Protein CbpA is described with a modular architecture comprising different repeat types and repetitions (Tulli et al., 2013), which is also true for protein CD3246 (van Leeuwen et al., 2021). The effect of the described sequence variants in the analyzed adherence proteins on strain virulence so far remains unclear. Different studies addressed divergent protein sequences of different adherence virulence factors such as CwpV, CbpA, or Cwp66 (Reynolds et al., 2011; Tulli et al., 2013; Zhou et al., 2022). However, these studies focused on the modular architecture of the protein (CwpV) instead of single amino acid deviations or investigated the effect of complete gene deletion or disruption (CbpA and Cwp66), which significantly altered cell adhesion, but also stress tolerance and antibiotic resistance in the case of Cwp66. Thus, the observed differences in the protein sequences of adherence virulence factors did not indicate a significant influence on bacterial colonization and the accompanying virulence of the corresponding clinical and non-clinical strains. Further, no clinical-related pattern in the various differences was observed.

The flagellar operon was represented by 41 CDSs in the *C. difficile-*specific protein sequence VFDB dataset. Here, the amount of the flagellar CDSs with over 90% query coverage to their VFDB reference sequence was used instead of sequence coverage of the individual proteins. Clinical and non-clinical counterparts showed identical coverages of predominantly 90% of the flagellar CDSs, whereas strains of ST11 only possessed 39% (Figure 1A). This coincided with our observation under the electron microscope and indicated that these two strains lack a flagellum. Sequence comparisons by BLAST+ blastp analysis (Camacho et al., 2009) between corresponding genomes revealed complete congruence for almost all strains and CDSs. Solely ST3 strains deviated in two protein sequences from each other (FlhG, FliN2) by maximal 0.81% (Figures 1A,B).

Concluding on the analysis of virulence factors between clinical and non-clinical strains, only a few differences in the protein sequences were observed that did not exhibit a connection to the clinical background of the strains. Therefore, a similar virulence would be expected for all strains, independent of their environmental or clinical origin. This conclusion coincided with the observations in the study by Zhou and colleagues (Zhou et al., 2021), where differences in the virulence factors (presence and sequence identity) between clinical and environmental strains were linked to the genotype but not bacterial origin.

### 3.3 Core/Pan genome analysis

**Figure 3:**
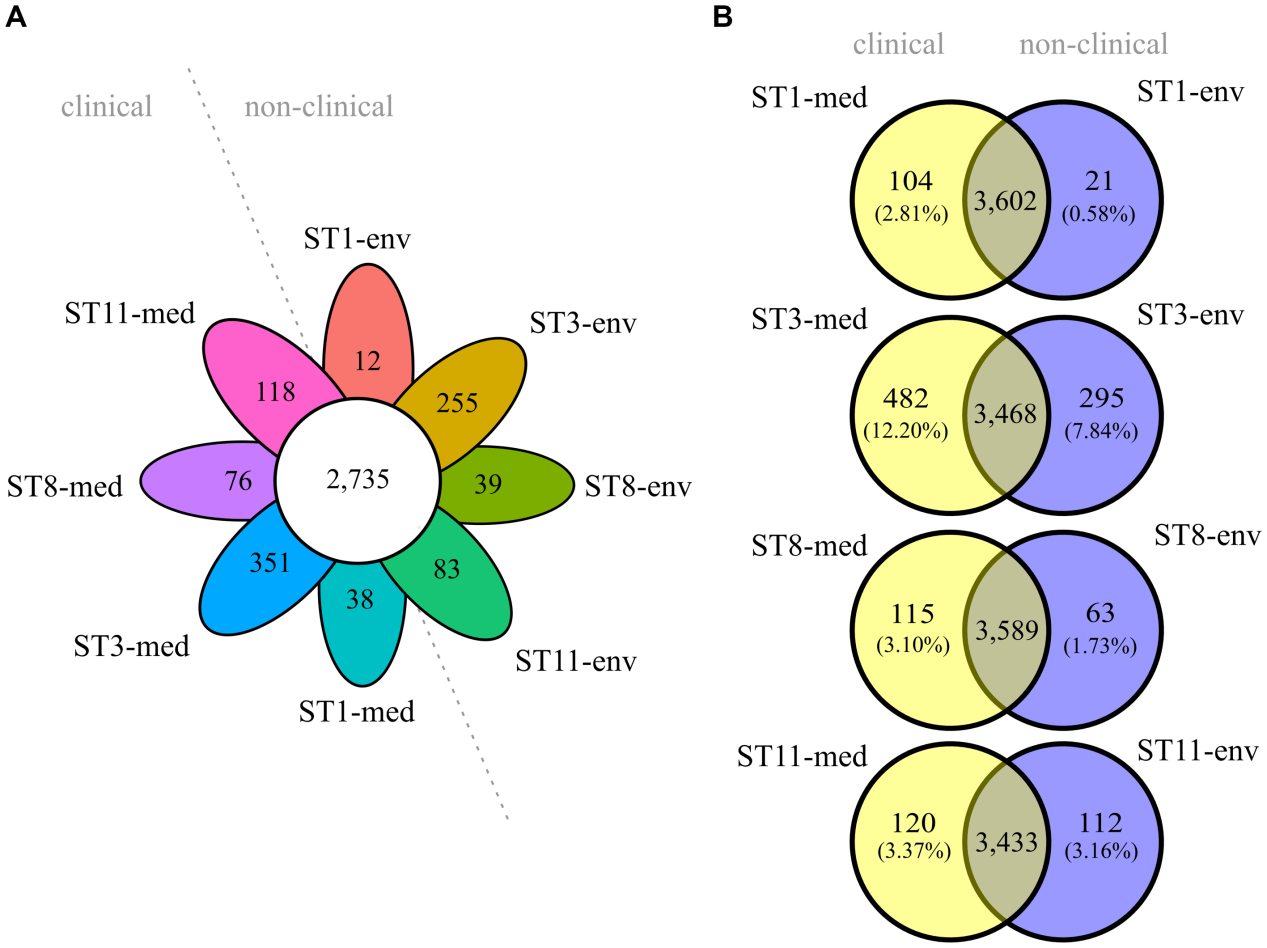
Core and accessory genome sizes of the analyzed *C. difficile* strains. Venn diagrams depicting the shared and unique genes among **(A)** all eight strains, or **(B)** pairwise between ST-corresponding clinical and non-clinical strain. The relative proportions of unique genes with regard to the total number of CDS per genome are indicated in parentheses below each absolute number of unique genes.

Overall core/pan genome analysis with all eight genomes resulted in 2,735 groups of core genes and varying numbers of unique genes between 12 and 351 (Figure 3A). A pan genome analysis of clinical strains grouped together and compared to the non-clinical strains demonstrated that the clinical strains possessed more accessory genes (634 vs. 400). However, none of these accessory genes were shared by all genomes of the four clinical strains. Similarly, the accessory genome of the non-clinical strains did not comprise genes present in all of the four genomes. Following, pairwise pan genome analyses of corresponding clinical and non-clinical strains verified the observation of more accessory genes in the genomes of the clinical strains. We calculated the relative proportions of unique genes per genome in relation to the total number of CDSs to take account of the different genome sizes (Figure 3B). Differences in accessory genome size between clinical and non-clinical strain ranged from 0.21% between ST11 strains to 4.36% between ST3 strains.

**Figure 4:**
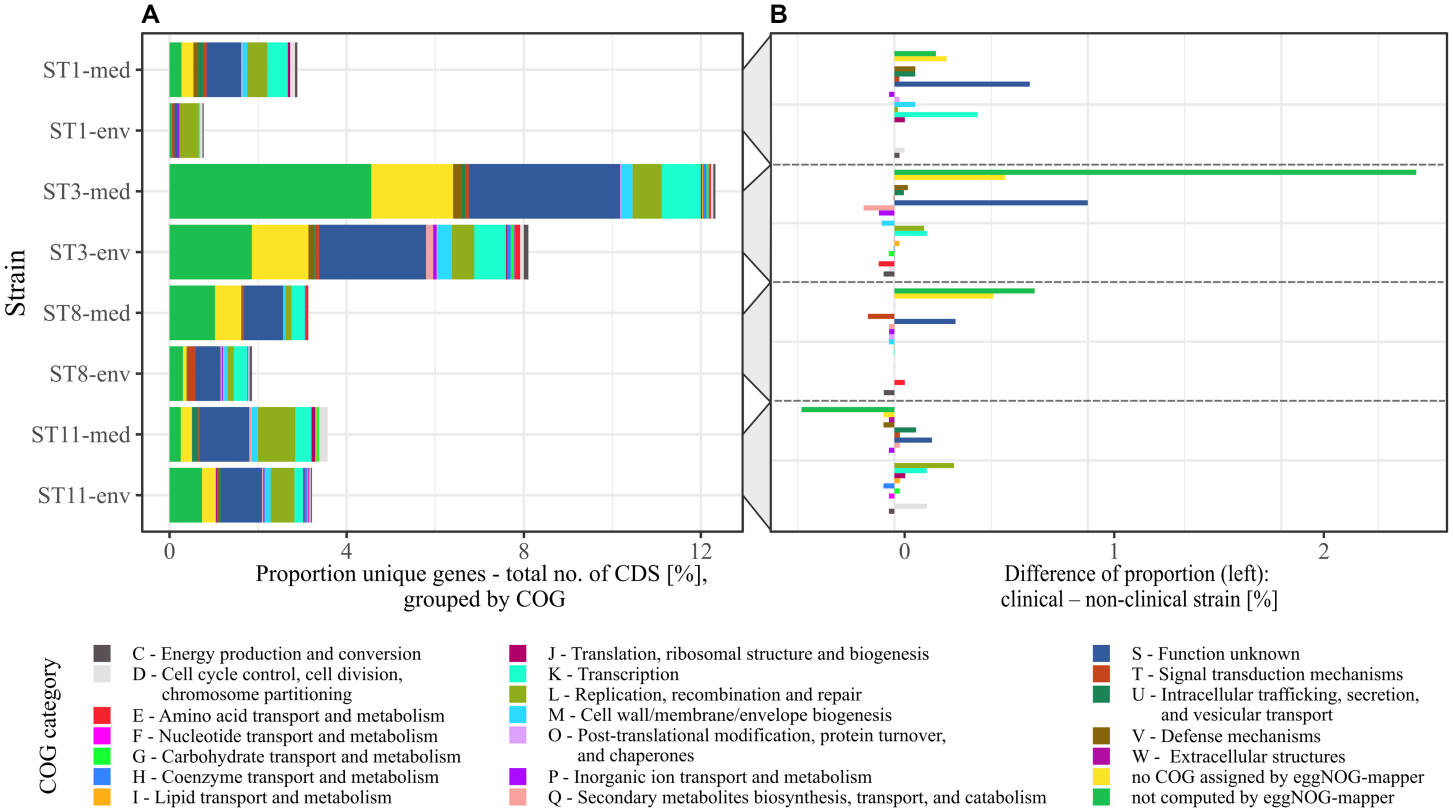
Relative abundance of COGs assigned to the unique genes from pairwise pan genome analyses. **(A)** The relative proportions of unique genes to total number of CDSs per genome in pairwise comparisons was transferred to their assigned COGs, which are designated with COG category name and function. **(B)** Individual COG proportions of unique genes of non-clinical strains were subtracted from the corresponding clinical strain to see if specific COGs are more frequent among accessory genes of a certain clinical background.

To elucidate genomic differences between clinical and non-clinical strains, unique genes were functionally classified into COGs with eggNOG-mapper (Cantalapiedra et al., 2021). Thereby, not all input genes could be classified, and several classified genes were not assigned to a specific COG. The number of un-classified or un-assigned genes varied between the genomes, but almost all these genes were annotated as hypothetical proteins. For better comparison, the relative abundance of assigned COGs in each accessory genome was calculated as described above (Figure 4A). This illustrated the previously determined differences in relative unique gene carriage within clinical and non-clinical strain pairs. Besides the noticeable numbers of unclassified and unassigned genes (hypothetical proteins), further bars representing the COG categories S (“Function unknown”), K (“Transcription”), L (“Replication, recombination and repair”) and also M (“Cell wall/membrane/envelope biogenesis”) were prominent to varying degrees in all strains, and seemed to be more abundant in the clinical strains. To further examine this, the differences in unique gene/COG proportions between corresponding clinical and non-clinical strains were determined (Figure 4B). This allowed identifying abundance trends of unique genes of specific COGs. The COG category S was the most abundant category and dominated in the clinical strains. Prokka-annotated functions (Seemann, 2014) of the genes assigned to COG category S were diverse and included for example phage-related proteins. Inspection of these genes for further potential virulence factors revealed the two genes encoding haemolysin XhlA and the virulence-associated protein E. Haemolysin XhlA and virulence-associated protein E are not associated with *C. difficile* virulence according to the data in VFDB (Liu et al., 2022), but are involved in the virulence of other bacteria such as *Clostridium chauvoei* (Thomas et al., 2021) and *Streptococcus suis* serotype 2 (Ji et al., 2016). Nevertheless, though *C. difficile* is not established as hemolytic pathogen, some evidence of hemolysis was recorded (Alkudmani, 2018). Influence of haemolysin XhlA and virulence-associated protein E on *C. difficile* virulence however has not been examined, yet, so that their virulence potential remains unknown. Both of these genes were present in ST3-env, while ST3-med and ST8-med possessed the haemolysin gene and ST11-med the virulence-associated protein E only. Thus, a direct relation to clinical background was not recorded for these specific genes.

The next highest differences were visible for COG categories L and K in particular, both being more abundant in three of the four clinical strains (ST1-/ST3-/ST11-med). Therefore, the accessory genomes of the clinical strains encoded more genes of unknown function, recombination- or transcription-associated activity than the genomes of the non-clinical strains. Interestingly, Lewis et al. identified accessory genes of these functions to correlate with higher strain virulence (Lewis et al., 2017). For example, the genes *rep* and *recF* encoding DNA helicase and DNA recombinase, and the gene *iap* encoding the lysozyme-like family protein were found in ST1 strains of high virulence, whereas they were absent in low-virulence ST1 strains. This gene presence/absence pattern was likewise true for our ST1 strains with respect to clinical background instead of *in vivo-*measured disease severity, implying a higher virulence potential of the clinical strain based on the accessory genome. Another study also identified accessory transcriptional regulators in hypervirulent strains of RT027 in contrast to the less virulent predecessor strain and pointed to the significance of these genomic accessories on strain virulence (Stabler et al., 2009). The increased proportion of transcriptional accessory genes compared to total CDS content along with the increased genome size/CDS carriage (Table 1) in the clinical strains might reflect adaptation to higher environmental variability (Parter et al., 2007). Noteworthy, Sebaihia et al. already pointed to the high amount of transcriptional regulators in *C. difficile* strain 630 and associated them with its potential ability to adapt to a rapidly changing environment (Sebaihia et al., 2006). Likewise, pan genome investigations on RT014 *C. difficile* strains isolated from farm pigs and infected humans did not compare clinical and non-clinical strains but also observed that the majority of accessory genes belonged to the functions of genetic or environmental information processing (Knight et al., 2017). Consequently, the clinical strains exhibited the genomic potential for faster reaction to changing environmental conditions such as emerging stress factors, which in turn affects survival as well as colonization and disease manifestation.

Although COG category M seemed noticeably abundant in the individual proportions (Figure 4A), it did neither exhibit a specific trend nor remarkable abundance in the proportional differences (Figure 4B). Contrary, category U (“Intracellular trafficking, secretion, and vesicular transport”) showed a noticeable trend toward genomes of clinical strains. Examination of the U-unique genes revealed that they were effectively restricted to clinical strains of ST1, ST3, and ST11, and thereby encoded only proteins involved in conjugal transfer of DNA, such as type IV secretory system components and relaxases/mobilization nuclease domain proteins. This is interesting in the context of horizontal gene transfer for fast adaptation to changing environmental conditions such as the presence of antibiotics (de la Cruz and Davies, 2000). Moreover, Brouwer et al. demonstrated the conjugative transfer of the PaLoc from a toxigenic to a non-toxigenic *C. difficile* strains, which turned the non-toxigenic strain into a toxin-producing one (Brouwer et al., 2013). Bacterial conjugation is mediated by cell-to-cell contact, which is eminently present in bacterial biofilms. Biofilm production and conjugation activity are therefore intertwined. This was already demonstrated in other bacteria such as *Escherichia coli* (Ghigo, 2001) and *Bacillus subtilis* (Lécuyer et al., 2018), where biofilm formation was shown to significantly impact conjugation efficiency. Taking this and the occurrence of conjugal accessory genes in clinical strains into account, the above identified sequence deviations in some adherence virulence factors could also be related to conjugal activity in the context of biofilm formation. This potential association has not been addressed in *C. difficile* so far, but would be worth considering in investigations on its virulence.

The only tendency to the non-clinical strains showed unique genes of the COG category P (“Inorganic ion transport and metabolism”), which was represented by only one gene per each non-clinical accessory genome encoding a cobalt transport protein, an ABC transporter transmembrane region, or a sodium sulfate symporter.

Conclusively, the comprehensive pan genome analysis in clinical and non-clinical strain comparisons established associations between clinical background and higher abundance of hypothetical proteins or proteins of unknown functions, and of genes linked to increased potential of conjugal and transcriptional activity. This trend is possibly linked to higher virulence, and in general can contribute to rapid physiological and evolutionary adaptation, which implies elevated virulence.

### 3.4 Prediction of ARGs

**Figure 5:**
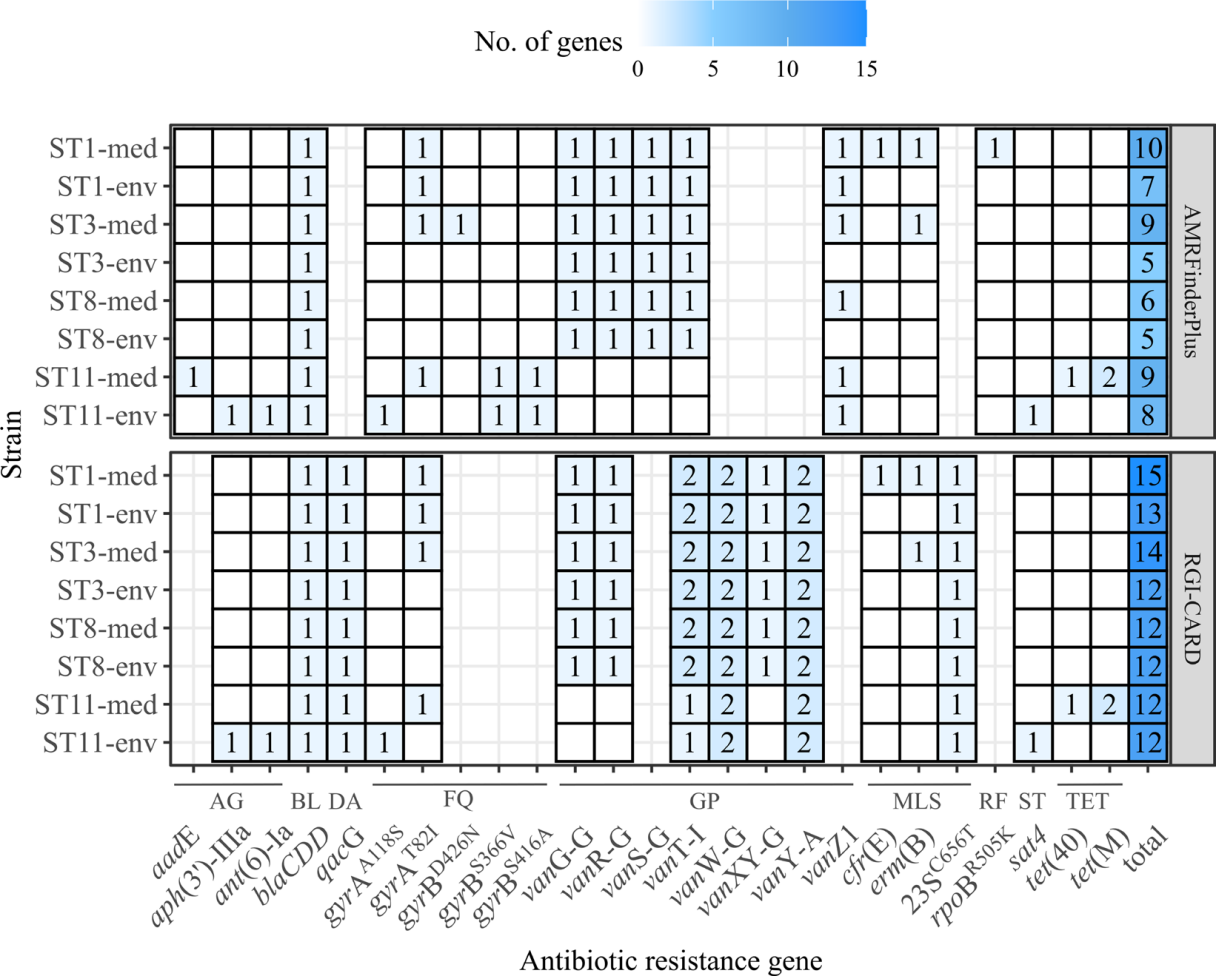
Predicted ARGs in the analyzed *C. difficile* strains. The number of identified ARGs and AR-conferring mutations as predicted with RGI-CARD (Alcock et al., 2023) and AMRFinderPlus (Feldgarden et al., 2021) were indicated by color and respective value, with white/no value meaning gene absence. Heatmap-tiles are missing for genes that were not part of the analysis tools. The total number of predicted ARGs for each program is additionally stated. ARGs are grouped according the associated antibiotic class: AG, aminoglycosides; BL, beta-lactams; DA, disinfecting agent and antiseptics; FQ, fluorquinolones; GP, glycopeptides; MLS, macrolides/lincosamides/streptogramins; RF, rifamycin; ST, streptothricins; TET, tetracyclines.

Antibiotic resistances are another crucial factor in *C. difficile* virulence, as they often allow *C. difficile* colonization and infection manifestation (Spigaglia, 2016). Further, ARGs can be linked to MGEs and contribute to the accessory genome (Sebaihia et al., 2006), which might partially explain the previously determined higher number of unique genes in the genomes of the clinical strains (Figure 3). We inspected all eight genomes for putative ARGs (including AR-conferring mutations) (Figure 5). Corresponding strains exhibited similar ARG patterns. Few genes were only identified in one strain or in multiple strains of the same ST or clinical background, respectively. For example, genes contributing to resistance against streptothricins (*sat4*) or aminoglycosides (*ant*(6)-Ia*, aph*(3′)-IIIa) were only identified in one non-clinical strain (ST11-env), while genes conferring resistance against macrolides (*erm*(B)), rifampin (*rpo*B^R505K^) or tetracyclines (*tet*(40)*, tet*(M)) were only detected in one clinical strain (ST11-med). Interestingly, tetracycline resistance has been linked to the emergence of ST11/RT078 strains as human pathogen (Dingle et al., 2019). However, the studies of Knetsch et al. (2014) and Zhou et al. (2021) identified the same ARGs in *C. difficile* strains of both environmental and clinical origin. Thus, a specific antibiotic resistance could not be linked to the clinical background. Regarding the ARG distribution with respect to the bacterial genotype, ST1 strains encoded most ARGs. Overall, clinical strains encoded one to four more ARGs than non-clinical strains.

### 3.5 Prediction of MGEs

**Figure 6:**
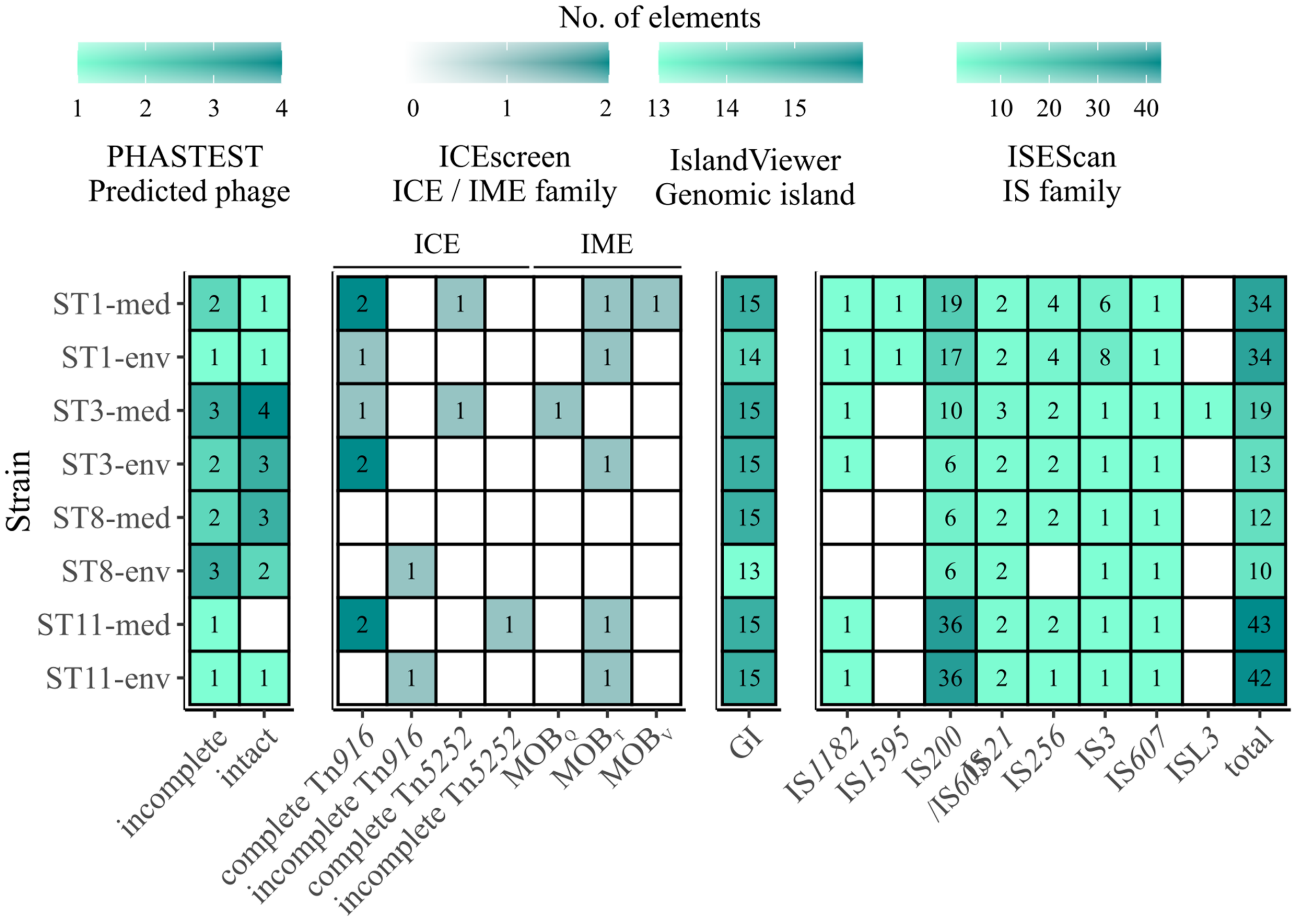
Predicted MGEs in the analyzed strains. The number of the analyzed MGEs prophages, integrative elements, GIs, and IS elements were indicated by color and the respective number, with white/no value meaning no prediction. Prophages are described as incomplete or intact as predicted by PHASTEST (Wishart et al., 2023). Integrative elements are categorized by the assigned superfamily and grouped into ICE and IME. Presence of IS elements is described in the context of the identified families, and additionally given as total number of IS elements.

All analyzed *C. difficile* genomes were investigated for MGEs. A plasmid family was solely present in ST11-med, representing the type repUS43 twice. Consequently, none of the observed ECEs was classified as plasmid, indicating another extrachromosomal type such as cryptic plasmids or prophages (Amy et al., 2018; Ramírez-Vargas et al., 2018). Thus, the genomes of all strains were analyzed for putative prophage regions. Contrary to the plasmid analysis, all ECEs besides the 7.6 kb element of ST3-env represented putatively intact prophages spanning the entire ECEs. Up to four incomplete or intact prophage regions were predicted per genome, except for ST11-med, which carried only one putative, intact prophage (Figure 6). Thereby, corresponding clinical and non-clinical strains showed comparable prophage carriage. This is in line with the study by Blau and Gallert (2024), where prophage analysis in 166 environmental *C. difficile* strains suggested a correlation between prophage carriage and strain genotype (ST/RT).

Integrative elements were detected in all strains except ST8-med (Figure 6). Two different types of elements, integrative and conjugative elements (ICEs) and integrative mobilizable elements (IMEs) were identified in both clinical and non-clinical strains. The two superfamilies Tn*916* and Tn*5252* represented ICEs, both observed as complete or incomplete modules without the ability of integration (“conjugation module”). The complete Tn*916* elements dominated, while incomplete Tn*916* and in−/complete Tn*5252* modules were likewise minor abundant. The ICE Tn*916* was already described in *C. difficile* and associated with antibiotic resistances, predominantly with *tet*(M) followed by *erm*(B) (Spigaglia et al., 2005, 2007; Dong et al., 2014), although the latter one is rather linked to other MGEs (Kartalidis et al., 2021). Consequently, this ICE is of interest in the context of spreading antibiotic resistances. Tn*5252* modules were restricted to clinical strains, while the incomplete Tn*916* modules were only found in non-clinical strains. Moreover, clinical ST1 and ST11 strains possessed three times more ICE modules than their non-clinical counterpart. Altogether, the ICE predictions could explain the presence of accessory genes with conjugal function (COG U) in the clinical strains described above, as they possessed elements that were missing in the corresponding non-clinical strains. Looking on IMEs, none were identified in ST8-strains, while all other isolates possessed IME modules of the families MOB_Q_, MOB_T_, and MOB_V_. MOB_T_ elements dominated with similar occurrence in clinical and non-clinical strains, whereas each of the other two IME modules occurred once, but only in clinical strains. Overall, IMEs were less common than ICEs. Similarly to ICEs, IMEs are potential carrier for ARGs such as *tet*(M) and, thus, are also involved in distribution of antibiotic resistances (López de Egea et al., 2023).

13 and predominantly 15 GIs were detected on each chromosome (Figure 6). These numbers exceeded the occurrences of the previously examined GI prophages and integrative elements and indicated the presence of other GI types. Depending on the ST, clinical strains possessed the same or a higher number of predicted GIs than the corresponding non-clinical strain. In addition to various GIs, we also examined IS elements and detected eight different IS families (Figure 6). The prevailing family was IS*200*/IS*605* with an incidence of six to 36 elements in the genomes of both ST11 strains. Elements belonging to families IS*21* and IS*256* occurred twice per genome on average, while family IS3 was present several times ST1 strain genomes and once in all other genomes. These IS families were identified to be potentially linked to ARGs and, consequently, might further contribute to ARG spread among *C. difficile* strains (Razavi et al., 2020). All other IS families were identified once per genome or not at all. Elements of type IS*1595* and ISL*3* were only found in ST11 or ST3-med strain genomes, respectively. Taken together, corresponding clinical and non-clinical strains exhibited similar IS patterns. Thereby, the total number of IS elements ranged between ten and 43 per genome, with clinical strains mostly possessing more IS elements (between one and six more elements) than the corresponding non-clinical strain. Further, general IS abundance was correlated to ST strain genomes, with ST8-strain genomes possessing the lowest and ST11-strain genomes the highest number of IS elements. Thus, regarding all above described MGEs, an overall trend of higher MGE carriage in clinical than in non-clinical strains was recorded, while no specific MGE was connected to clinical background.

### 3.6 Pairwise genome comparisons with implementation of preceding analyses

**Figure 7:**
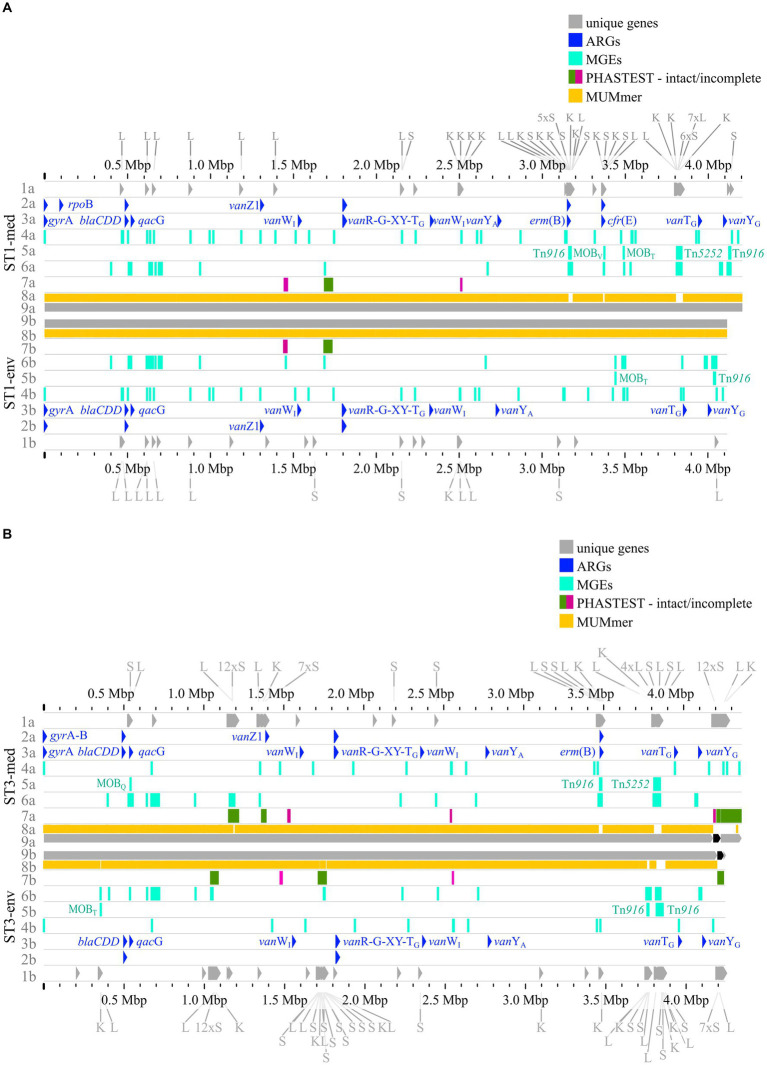
Pairwise genome comparisons complemented with the predicted ARGs, MGEs, and accessory genes. Genome comparisons of (A) ST1-strains and (B) ST3-strains are depicted with different tracks for each visualized feature in the clinical strain at the top (track letter a) and non-clinical strain at the bottom (track letter b). The tracks represent: 1 unique genes with genes assigned to COG S, K, L highlighted, and multiple genes of the same COG grouped together if necessary for better visibility, 2 AMRFinderPlus (Feldgarden et al., 2021) predicted ARGs, 3 RGI+CARD (Alcock et al., 2023) predicted ARGs, 4 predicted IS elements, 5 predicted integrative elements labelled with assigned superfamily, 6 predicted GIs, 7 prophage prediction with completeness color-coded according to PHASTEST (Wishart et al., 2023), 8 MUMmer alignment (Kurtz et al., 2004), 9 replicons.

**Figure 8:**
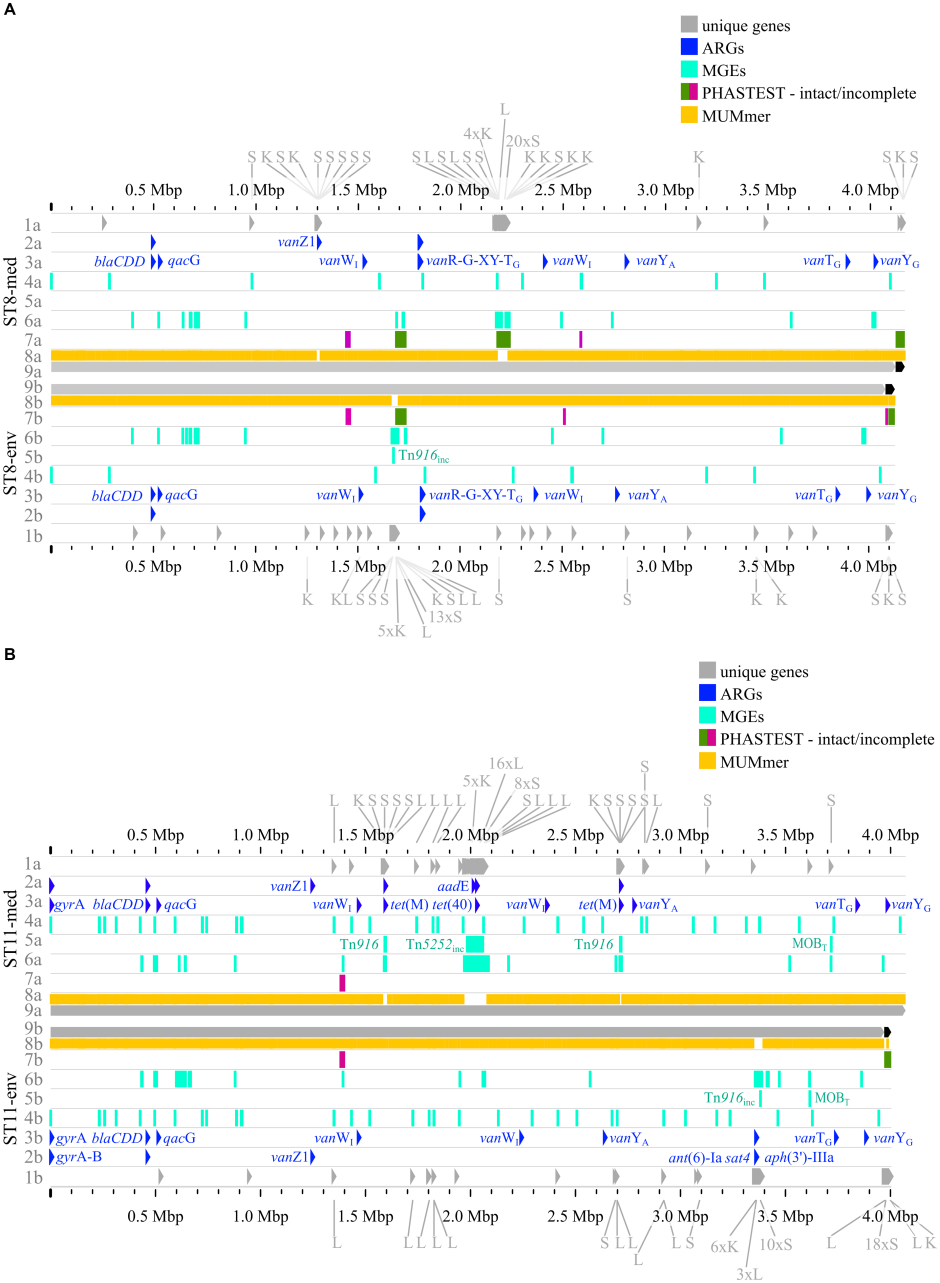
Pairwise genome comparisons complemented with the predicted ARGs, MGEs, and accessory genes. **(A)** ST8-strains, and **(B)** ST11-strains are depicted with different tracks for each visualized feature in the clinical strain at the top (track letter a) and non-clinical strain at the bottom (track letter b). The tracks represent: 1 unique genes with genes assigned to COG S, K, L highlighted, and multiple genes of the same COG grouped together if necessary for better visibility, 2 AMRFinderPlus (Feldgarden et al. 2021) predicted ARGs, 3 RGI+CARD (Alcock et al. 2023) predicted ARGs, 4 predicted IS elements, 5 predicted integrative elements labelled with assigned superfamily, 6 predicted GIs, 7 prophage prediction with completeness color-coded according to PHASTEST (Wishart et al. 2023), 8 MUMmer alignment (Kurtz et al. 2004), 9 replicons.

The previous analyses of accessory genes, ARGs, and MGEs showed differences between clinical and non-clinical strains. All these results were combined with MUMmer alignments (Kurtz et al., 2004) and together put into genomic context in pairwise genome alignments (Figures 7, 8). This representation revealed connections between the various analyzed elements based on co-occurrence. First, the MUMmer alignments again demonstrated the higher abundance of unique genes in the clinical strains, as they exhibited more alignment gaps that corresponded to missing regions in the corresponding non-clinical strain. Consequently, the majority of the unique genes were found next to each other in clusters. Mapping of the predicted MGEs and ARGs illustrated that they resided at the same genomic positions in the corresponding strains. MGEs and ARGs that were only present in one of the two compared strains (mostly the clinical strain) often occurred together. The genome of strain ST1-med (Figure 7A) possessed the ARG *erm*(B) that resided within ICE Tn*916*. This conjunction was observed in ST3-med genome as well (Figure 7B) and supported the already mentioned connection between Tn*916* elements and ARG *erm*(B) (Spigaglia et al., 2005, 2007). ARG *tet*(M) in the ST11-med genome exhibited the presumed connection with Tn*916* (Figure 8B) (Dong et al., 2014). ST11-med further possessed ARGs *tet*(40) and *aad*E, which occurred within an incomplete Tn*5252* element (Figure 8B). In contrast, the non-clinical ST11 strain carried three ARGs (*ant*(6)-Ia*, sat4, aph*(3′)-IIIa) close to each other and to an incomplete Tn*916* element, thus located outside of this predicted ICE region (Figure 8B). However, the GI prediction identified a larger mobile region than determined for the incomplete Tn*916* that included the three ARGs. The mobile region was confirmed by an alignment gap and a cluster of unique genes of similar size. The occurrence of the ARGs *ant*(6)-Ia, *sat*4, and *aph*(3′)-IIIa as a resistance cassette was already observed in genomes of ST13 and ST49 *C. difficile* strains (RT014, clade 1) from porcine origin (Knight et al., 2017) and in genomes of ST11 strains (RT126 and RT078) from environmental sources (Blau et al., 2023). Both studies did not address a connection between this resistance cassette and a MGE, although the latter one hypothesized the possibility of genetic transmission (Blau et al., 2023). Another ARG, *qac*G of the ST3-med genome (Figure 7B), was located within a predicted GI and next to a complete IME of family MOB_Q_. All further *qag*G genes and other ARGs were not colocalized with MGEs. However, some of these ARGs resided in close proximity to predicted MGEs, such as the remaining *qac*G genes (Figures 7A,B, 8A,B), *cfr*(E) in the ST1-med genomes (Figure 7A), or *van*T-G in ST1 and ST3 strain genomes (Figures 7A,B). Connections between these colocalized ARGs and MGEs are speculative but might still be interesting for further investigations regarding dissemination of antibiotic resistances. For instance, ARG *cfr*(E) was also found within an undescribed MGE in genomes of RT027 *C. difficile* strains from Mexico (Stojković et al., 2019).

The predicted prophage regions, integrative elements, and GIs accounted for the majority of the genomic differences between corresponding strains in the form of alignment gaps (Figures 7A,B, 8A,B). Hargreaves et al. likewise already pointed out that major regions of genome divergence in *C. difficile* strains from estuarine samples belonged to the MGE-type transposons (Hargreaves et al., 2015). The unique genes in these regions largely belonged to COG categories S, K, and L (Figures 7A,B, 8A,B), which showed the association of these unique genes to MGEs. This is especially interesting for the COG category S of “Unknown function,” which implied their involvement in the function of the respective MGE or an encoded accessory function that is potentially relevant for strain virulence. Accessory genes of COG category S with virulence potential were already identified during the pan genome analysis, which encoded haemolysin XhlA and the virulence-associated protein E. Within this genome comparison analyses, these potential virulence factors were now associated to MGEs and, thus, might be transferrable between cells, which sheds another light on these potential virulence factors. Many of the unique genes that were only annotated as hypothetical proteins belonged to the MGE-associated clusters, which indicates involvement in MGE-related functions.

## 4 Conclusion

The comprehensive genome analyses and comparisons of corresponding clinical and non-clinical *C. difficile* strains revealed genomic patterns associated with clinical background. No distinct differences in virulence factors known to be crucial for *C. difficile* virulence, such as toxins and proteins involved in adherence, sporulation, exoenzymatic reaction and motility were detected. Thus, corresponding strains possessed the same fundamental virulence equipment, which suggested same virulence regardless of the clinical/non-clinical background. Pan genome analysis revealed that clinical strains possessed a larger accessory genome. Assignment of the unique genes to functional clusters demonstrated the trend in clinical strains with more unique genes previously annotated as hypothetical proteins or functionally assigned to COG categories S, K, L, and U. Such trend of those accessory genes/functions is linked to higher virulence and enables the strain to rapidly respond to changing environmental conditions such as emerging stress, which supports bacterial survival, colonization, and disease manifestation. Further analyses predicted various ARGs and MGEs. No particular ARG/MGE was specifically linked to clinical background, but the overall trend of more ARGs and MGEs in clinical strains was observed. Results from pan genome, ARG, and MGE analyses together in genome alignments revealed conjunctions between specific ARGs and MGEs. The genome comparisons further demonstrated that genomic differences between clinical and non-clinical strains mainly originated from MGEs. This also included the majority of unique genes with higher abundance in clinical strains that were assigned to COG categories with connection to increased virulence and faster physiological reaction capacity. Consequently, these trends suggested adaptations of the clinical strains by gene acquisition that might manifest in higher strain virulence. This should be further investigated to elucidate *C. difficile* virulence and progression, especially in the context of clinical and non-clinical strain comparison. Therefore, future investigations are advised to incorporate non-clinical strains in comparative analyses for a comprehensive understanding of *C. difficile* virulence development. These findings also highlight the importance of MGEs for *C. difficile*, since they seem to be involved not only in the dissemination of ARGs or virulence factors but also impact virulence in another way. We further advise to examine genomic analyses in whole-genome context to reveal conjunctions between the various elements.

## Data availability statement

Genome data of the bacterial strains is deposited at NCBI (https://www.ncbi.nlm.nih.gov/) under following GenBank accession numbers: *Clostridioide difficile* strain TS3_3 (ST1-env) under CP134872, strain B1_2 (ST3-env) under CP132141-3, strain J2_1 (ST8-env) under CP134690-1, strain MA_1 (ST11-env) under CP132139-40, DSM28196 (ST1-med) under CP012320, SC084-01-01 (ST3-med) under CP132146-8, SC083-01-01 (ST8-med) under CP132144-5, and DSM29747 (ST11-med) under CP019864.

## Author contributions

MS: Conceptualization, Data curation, Formal analysis, Methodology, Validation, Visualization, Writing – original draft, Writing – review & editing. TR: Validation, Writing – review & editing. JO: Validation, Writing – review & editing. RD: Investigation, Project administration, Validation, Writing – review & editing. AP: Supervision, Validation, Writing – review & editing.

## Footnotes

^1^ https://github.com/rrwick/Porechop

^2^ https://github.com/rrwick/Filtlong

^3^ https://github.com/boykebunk/genomefinish

^4^ https://inkscape.org/de/
