# Supplementary material for: Comparative genome analyses of clinical and non-clinical Clostridioides difficile strains
Source: Front Microbiol. 2024 Jun 27;15:1404491. doi: 10.3389/fmicb.2024.1404491 (PMC11238072; doi:10.3389/fmicb.2024.1404491)
Supplement: Supplementary file 1 [file Presentation_1.pdf]

Supplementary file S1

Isolation protocols of the non-clinical *C. difficile* strains

BHIS - supplemented Brain Heart Infusion Broth (BHIS; supplemented with 0.5% yeast extract, 0.05% L-cysteine, 0.0001% Na-resazurin, purged with nitrogen)

C. difficile strain MA_1 – antibiotic-based isolation

Isolation was based on the study of Dharmasena and Jiang (Dharmasena and Jiang, 2018). 25 g of environmental sample were suspended in 100 ml PBS, and solid particles removed with a filter paper. Cells were repeatedly washed via centrifugation before inoculated into the enrichment medium BHIB-YE-CYS-MN-T medium (Brain Heart Infusion Broth, 0.5% yeast extract, 0.05% L-cysteine, 0.1% sodium taurocholate, moxalactam-norfloxacin (CDMN, Oxoid Deutschland GmbH, Wesel, Germany) and following the described enrichment procedure.

C. difficile strain J2_1 – antibiotic-free isolation

Isolation was performed under strict anaerobic conditions. The environmental sample was suspended in 30 ml PBS via slight shaking and soaking at room temperature for five hours, and 1 ml was transferred to a reaction tube without withdrawing solid particles. The sample was pasteurized at 80°C for 10 minutes, and 100 µl undiluted or 1:2 diluted aliquots were plated on solid BHIS 1.5% agar supplemented with 0.1% taurocholic acid to promote *C. difficile* spore germination (Sorg and Sonenshein, 2008), some plates additionally contained the antibiotic moxalactam norfloxacin (CDMN, Oxoid). Plates were incubated at 37°C until colonies were visible, and strain J2_1 was isolated from an antibiotic-free plate.

C. difficile strain B1_2 – antibiotic-based isolation

Isolation was performed under strict anaerobic conditions. The environmental sample was treated as described above for the isolation of *C. difficile* strain J2_1 until plating of sample aliquots. 100 µl aliquots of 1:2, 1:10, and 1:100 dilutions were plated on plates and incubated as described for strain J2_1. Strain B1_2 was isolated from an antibiotic-containing plate.

C. difficile strain TS3_3 – antibiotic-free isolation

Isolation was performed under strict anaerobic conditions, and enrichment was done in a minimum amino acid-defined medium (Karasawa et al., 1995; Yamakawa et al., 1996) to metabolically select for *C. difficile*. The medium based on the defined medium of Yamakawa et al., 1996 (Yamakawa et al., 1996) with 1x amino acid composition and omitting Glucose, and was additionally supplemented with Na_2SeO_3, $Na_2MoO_4 \cdot 2\ H_2O$, and $Na_2WO_4 \cdot 2\ H_2O$ at the final concentration of 0.1 mg/L and Na-resazurin 0.0001% as anaerobic indicator. The medium was purged with N_2/CO_2 (80%/20%) and further reduced if necessary by dropwise addition of a Na_2S solution until the resazurin indicator turned colorless. The pH was adjusted to 7.2 if necessary. The medium was finally sterilized with a Filtropur S 0.2 µm filter (SARSTEDT AG & Co. KG, Nürnbrecht, Germany) while kept anaerobic. A scoop of the environmental sample was dissolved in 30 ml PBS via slight shaking and soaking at room temperature for five hours, 2 ml aliquots were briefly centrifuged to pellet solid particles and 1.5 ml of the supernatant transferred to new tubes for pasteurization at 80°C for 10 minutes. Subsequently, spores were briefly pelleted and incubated at 37°C for 45 minutes in an equal volume of PBS supplemented with 0.1% taurocholic acid and 1.3 mM glycine to promote *C. difficile* spore germination (Sorg and Sonenshein, 2008). This approach was used to inoculate 250 ml of enrichment medium, and incubated at 37°C for three days. 100 µl aliquots and 2 ml

aliquots concentrated to 100 µl were plated on standard solid BHIS 1.5% agar and incubated at 37°C until colonies were visible.

References

Dharmasena, M., and Jiang, X. (2018). Improving culture media for the isolation of *Clostridium difficile* from compost. *Anaerobe* 51, 1–7. doi: 10.1016/j.anaerobe.2018.03.002

Karasawa, T., Ikoma, S., Yamakawa, K., and Nakamura, S. (1995). A defined growth medium for *Clostridium difficile*. *Microbiology* 141, 371–375. doi: 10.1099/13500872-141-2-371

Sorg, J. A., and Sonenshein, A. L. (2008). Bile Salts and Glycine as Cogerminants for *Clostridium difficile* Spores. *Journal of Bacteriology* 190, 2505–2512. doi: 10.1128/JB.01765-07

Yamakawa, K., Karasawa, T., Ikoma, S., and Nakamura, S. (1996). Enhancement of *Clostridium difficile* toxin production in biotin-limited conditions. *Journal of Medical Microbiology* 44, 111–114. doi: 10.1099/00222615-44-2-111
